# Generation and characterization of a stable cell line persistently replicating and secreting the human hepatitis delta virus

**DOI:** 10.1038/s41598-019-46493-1

**Published:** 2019-07-10

**Authors:** Yi Ni, Zhenfeng Zhang, Lisa Engelskircher, Georg Verch, Thomas Tu, Florian A. Lempp, Stephan Urban

**Affiliations:** 1 Department of Infectious Diseases, Molecular Virology, University Hospital Heidelberg, Heidelberg, Germany; 2 German Center for Infection Research (DZIF), partner site Heidelberg, TTU Hepatitis, Heidelberg, Germany

**Keywords:** Hepatitis B virus, Viral transmission

## Abstract

Human hepatitis delta virus (HDV) causes the most severe form of viral hepatitis. Approximately 15–25 million people are chronically infected with HDV. As a satellite virus of the human hepatitis B virus (HBV), HDV uses the HBV-encoded envelope proteins for egress from and *de novo* entry into hepatocytes. So far, *in vitro* production of HDV particles is restricted to co-transfection of cells with HDV/HBV encoding cDNAs. This approach has several limitations. In this study, we established HuH7-END cells, which continuously secrete infectious HDV virions. The cell line was generated through stepwise stable integration of the cDNA of the HDV antigenome, the genes for the HBV envelope proteins and the HBV/HDV receptor NTCP. We found that HuH7-END cells release infectious HDV particles up to 400 million copies/milliliter and support virus spread to co-cultured cells. Due to the expression of NTCP, HuH7-END cells are also susceptible to *de novo* HDV entry. Virus production is stable for >16 passages and can be scaled up for preparation of large HDV virus stocks. Finally, HuH7-END cells are suitable for screening of antiviral drugs targeting HDV replication. In summary, the HuH7-END cell line provides a novel tool to study HDV replication *in vitro*.

## Introduction

Hepatitis delta virus (HDV), first described in 1977^1^, is a viroid-like pathogen and belongs to the genus *Deltavirus*. Recently, HDV-like sequences have been found in snakes^2^ and ducks^3^. As a satellite virus of hepatitis B virus (HBV), HDV requires HBV for propagation and spread in the human liver. Co-infection with HBV and HDV (occurring in 15–25 million chronic HBV infected individuals) leads to a faster progression to liver cirrhosis^4^ and a higher risk of hepatocellular carcinoma compared to chronic infection with HBV alone. There is currently no specific treatment for HDV infection, however two novel drugs (Myrcludex B/Bulevirtide and Lonafarnib) have recently entered phase III registration trials^5^. Off-label use of IFNα showed moderate viral response rates in HDV-infected patients, but viral relapses were frequently observed after stopping treatment. Thus, further research into this pathogen is needed to develop and test new antiviral agents.

Similar to plant viroids, HDV consists of a circular RNA genome, which replicates by rolling circle amplification generating genomic and antigenomic viral RNA strands. RNAs of both polarities have ribozyme activity required for self-cleavage of the respective cognate strand^6^. The circular HDV RNAs (1672–1697 nt in length depending on the genotype^7^) are highly self-complementary (approximately 74% base pairing) and fold into unbranched rod-like structures which associate with the hepatitis delta antigen to form ribonucleoprotein (RNP) complexes^6,8–10^. Since the extracellular HDV virions are composed of an envelope derived from the HBV proteins, both HBV and HDV utilize the receptor sodium taurocholate co-transporting polypeptide (NTCP) for entering into hepatocytes^11,12^. Following entry, the HDV RNP complex is transported to the nucleus, where viral replication is initiated by cellular RNA polymerases. Replication of HDV RNA is recognized by cellular melanoma differentiation antigen 5(MDA5) thereby inducing IFNs beta and lambda^13^.

HDV encodes a single protein called hepatitis delta antigen (HDAg) that is expressed in a small and a large form (S-HDAg and L-HDAg). They are required for viral RNA replication and egress of particles respectively. The production of L-HDAg is regulated by a cellular RNA-specific adenosine deaminase ADAR1, which mutates the stop codon of the HDAg ORF on the anti-sense HDV RNA. This mutation alters the stop codon into a tryptophan codon, thereby extending the S-HDAg into the 19aa-longer L-HDAg. The L-HDAg becomes C-terminally prenylated within this elongated sequence by a host farnesyl transferase^14^, complexes with HDV genomic RNA, and the new RNP complex is packaged into HBV envelope proteins, which is subsequently secreted by the infected cells.

To study HDV secretion *in vitro*, current systems rely on co-transfection of hepatic cells with HDV- and HBV envelope-expressing plasmids^15^ or the HepNB2.7 cell line that supports the release of progeny HDV upon infection^16^. While the HBV field has developed several virus-replicating cell lines (such as HepG2.2.15^17^ and HepAD38^18^, which have been used for virus production, investigation of the viral replication cycle and identification of antiviral drug candidates), there is currently no equivalent stable cell line that supports continuous HDV replication and secretion of infectious HDV particles.

In the current study, we describe a stable cell line called HuH7-END that allows high levels of HDV secretion and can be used for large-scale virus preparation. Since it supports continuous replication of HDV from an integrated cDNA it is also useful as a screening platform to determine the effect of compounds affecting later stages of HDV replication and release. Additionally, the HuH7-END cell line can be used to identify compounds that affect HBsAg (hepatitis B surface antigen) secretion for HBV drug screening approaches. In this paper, we characterize the virological aspects of this system that simplifies many aspects of HDV research.

## Results

### Establishment of the HuH7-END cell line

**Figure 1:**
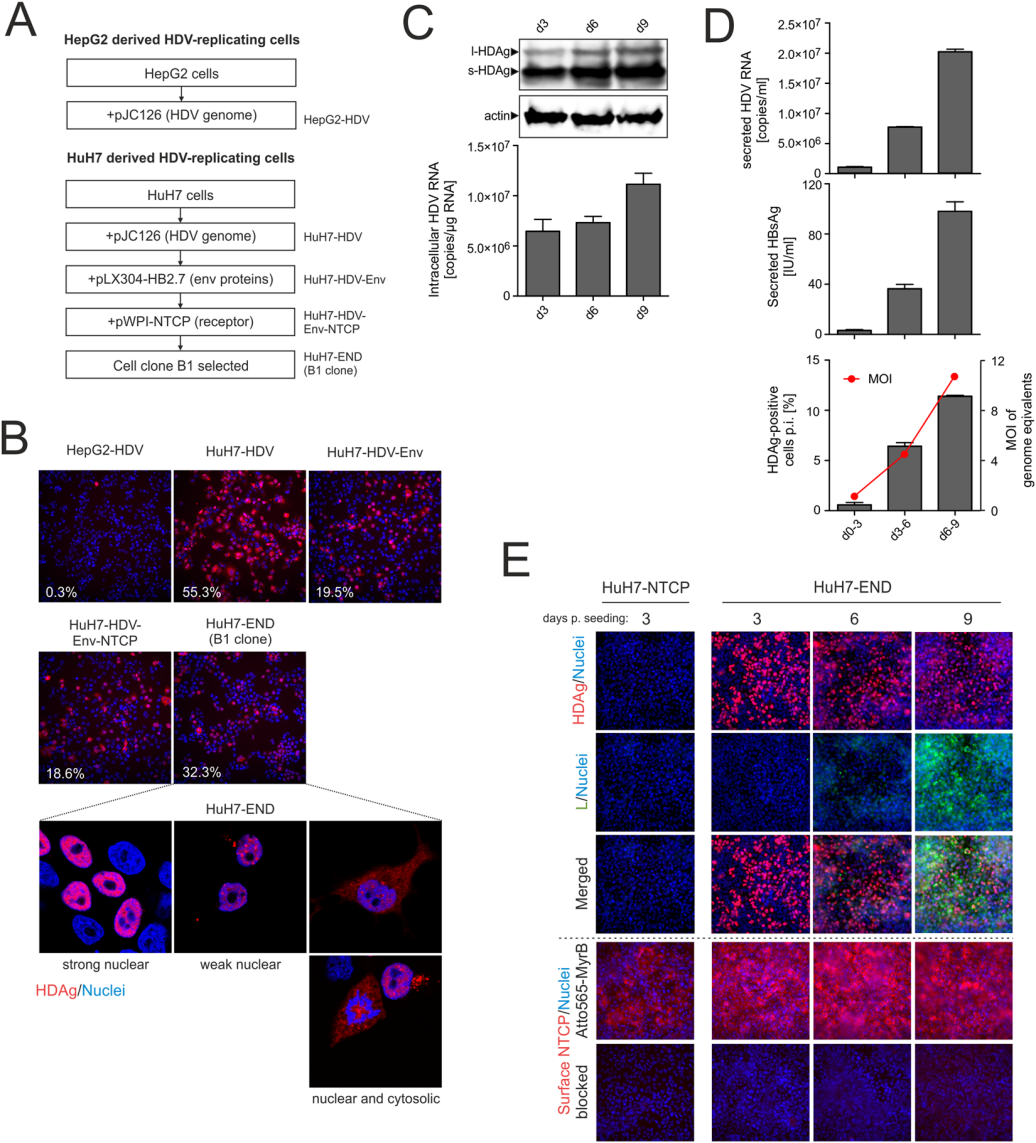
Establishment and characterization of HDV replicating cell lines. (**A**) Generation scheme of a HepG2-derived cell line stably expressing HDV (HepG2-HDV) and HuH7 cells expressing HDV (HuH7-HDV), HDV and the HBV envelope proteins (HuH7-HDV-Env) and HDV, HBV envelope proteins plus human NTCP (HuH7-HDV-Env-NTCP). Finally, a single cell clone of HuH7-HDV-Env-NTCP cells was named as HuH7-END. (**B**) Characterization of the HDV-replicating cell lines described above: HepG2-HDV, HuH7-HDV, HuH7-HDV-Env cells (top row) and HuH7-HDV-Env-NTCP (pool) and clone B1 (middle row) were seeded and stained for HDAg at day 1 post seeding (HDAg in red). Confocal image analyses of HDAg-stained single cells from the HuH7-END cells (lower row) revealed three distinguishable patterns of the subcellular location of HDAg. The numbers given in the pictures indicate the percentage of HDAg-positive cells. (**C**) Western blot analysis of HDAg (top) and quantification of intracellular HDV RNA (below) of HuH7-END cells at d3, d6 and d9 post seeding. (**D**) Analyses of particle secretion of HuH7-END cells. Cell culture medium of the indicated time frames were harvested and quantified for secreted HDV RNA (top), HBsAg (middle). The secreted infectious HDV is determined by the number of HDAg-positive HuH7-NTCP cell post-infection (bottom). For the latter the calculated MOI is shown as the red line. (**E**) Characterization of the HuH7-END cell line with respect to HDAg, HBV L-protein and expression of NTCP. HuH7-NTCP cells (d3 post seeding) and HuH7-END cells at d3, d6 and d9 post seeding were analyzed by IF for HDAg (row 1), HBV L-protein (row 2) and the merged pictures (row 3, HDAg in red, L protein in green). Nuclei were counterstained with Hoechst. Surface NTCP is labelled using 100 nM Atto565-labelled MyrB (row 4). Pretreatment of cells with 2 µM unlabelled MyrB (row 5) was used to ensure specific NTCP labelling.

To generate cell lines allowing steady intracellular replication of HDV, two commonly used human hepatic cell lines HuH7 and HepG2 were transfected with pJC126, a plasmid harboring a 1.1-fold cDNA copy of the HDV antigenome^19^ and a neomycin resistance gene. After selection with G418, the pool of cell clones (referred to as HuH7-HDV and HepG2-HDV, Fig. 1A) were expanded and analyzed by HDAg-specific immunofluorescence. Approximately 55% of HuH7-HDV but only 0.3% of HepG2-HDV cells stained positive for HDAg (Fig. 1B), despite pJC126 initiating HDV replication^19^ (and therefore HDAg-expression). This result suggested that HepG2-HDV cells somehow down-regulates HDAg (which may be related to their innate immune competence^13^). We therefore proceeded to use HuH7-HDV cells to continue with further engineering.

Since HBV envelope proteins enable secretion of HBV subviral particles and support envelopment of the HDV RNPs, we stably transduced the HuH7-HDV with a lentiviral vector harboring a 2.7-kb HBV fragment encoding the HBV envelope proteins under control of their native promoter/enhancer^20^. Such a construct has also been successfully implemented in the fully replication-competent cell line HepNB2.7^16^. Following selection with blasticidin, the cell pool (referred to as HuH7-HDV-Env) secreted HBsAg as well as infectious HDV virions, as shown by quantitative HBsAg ELISA and infection of HepG2-NTCP cells using cell culture supernatants (Supplemental Fig. 1).

HuH7-HDV-Env cells cannot amplify HDV via the extracellular route as they lack NTCP the essential receptor for *de novo* entry. To enable receptor-mediated HDV entry, we transduced HuH7-HDV-Env cells with an NTCP-encoding lentiviral vector^11^. After selection of a cell pool (referred to as HuH7-HDV-Env-NTCP) single colonies were isolated, expanded and characterized for the HBsAg secretion as well as HDV RNA replication. One clone B1 was selected based on its continuous high-level secretion of HDV RNA and HBsAg and referred to as HuH7-END (abbreviation of **E**nvelope, **N**TCP and H**D**V) (Fig. 1A).

During stepwise engineering of the HuH7-END cells, we monitored intracellular HDAg expression in the intermediate cell lines. About 18–55% of the cells stained positive for HDAg. Interestingly, after clonal isolation, the HuH7-END cell clone displayed strong HDAg expression in only approximately 30% of cells (Fig. 1B). To analyze this heterogeneity further, we visualized HDAg in the HuH7-END cells by confocal microscopy. A subpopulation of HuH7-END cells maintained very low or undetectable HDAg. This lack of HDAg expression in a subpopulation of stably transduced cells is consistent with the observation previously reported in HuH7-D12 cell line^21^.

### Characterization of the HuH7-END cells

To analyze HDAg expression, HDV RNA replication and editing, we measured HDAg by Western blot and HDV RNA by qPCR at day 3, 6 and 9 post seeding. L-HDAg could be detected at all time-points at a constant ratio to S-HDAg (Fig. 1C), indicating that RNA editing occurs and does not change significantly during cultivation of cells. Moreover, constant levels of intracellular HDV RNA were detected at any time point during cultivation, indicating continuous RNA replication.

We further measured secreted HDV RNA and HBsAg levels, and used the culture supernatant to infect HuH7-NTCP cells. All viral readouts (HDV RNA, HBsAg and the number of HDAg positive recipient cells infected by the supernatant) reached the highest levels around d9 post seeding (Fig. 1D). This delayed peak in secreted HDV RNA and infectious virions (which coincided with the onset of HBsAg secretion) contrasts to the relatively constant level of intracellular HDV RNA. This suggests that HBsAg secretion is the rate-limiting step of HDV virion production in this system.

HDAg-positive cells were readily detectable between d3 and d9 post seeding by IF (immunofluorescence staining), consistent with the results from the Western blots detecting intracellular HDAg. In contrast, The HBV L protein (stained with the mAb MA18/7) followed a much slower expression kinetics and became detectable earliest at d6 and more prominent at d9 post seeding (Fig. 1E, upper panels). This potentially indicates that cells more efficiently express HBV envelope protein while in a cellular steady state.

To confirm the surface expression of the HDV receptor NTCP, we took advantage of an Atto-565 labelled variant of the HBV/HDV entry inhibitor Myrcludex B (MyrB) for fluorescent labelling of surface NTCP receptor^22^. Compared to previously reported HuH7-NTCP cells^11^, HuH7-END cells displayed higher surface NTCP levels. The specificity of NTCP staining was confirmed by competition with non-labelled MyrB (Fig. 1E, lower panels).

HuH7-END cells displayed three distinct subcellular HDAg location patterns (Fig. 1B, lowest row). The majority of the HDAg-positive cells showed an intense staining of HDAg within the nucleus. The second type of staining was also nuclear but showed a weaker and punctate distribution. Both patterns have been reported previously in HuH7-D12 cells^21^. The third pattern displayed HDAg signals in both nuclei and cytosol. This staining has previously been reported^23^ and was often observed in cells with condensed chromatin, indicating ongoing cell division.

### Continuous and large-scale production of infectious HDV by HuH7-END

**Figure 2:**
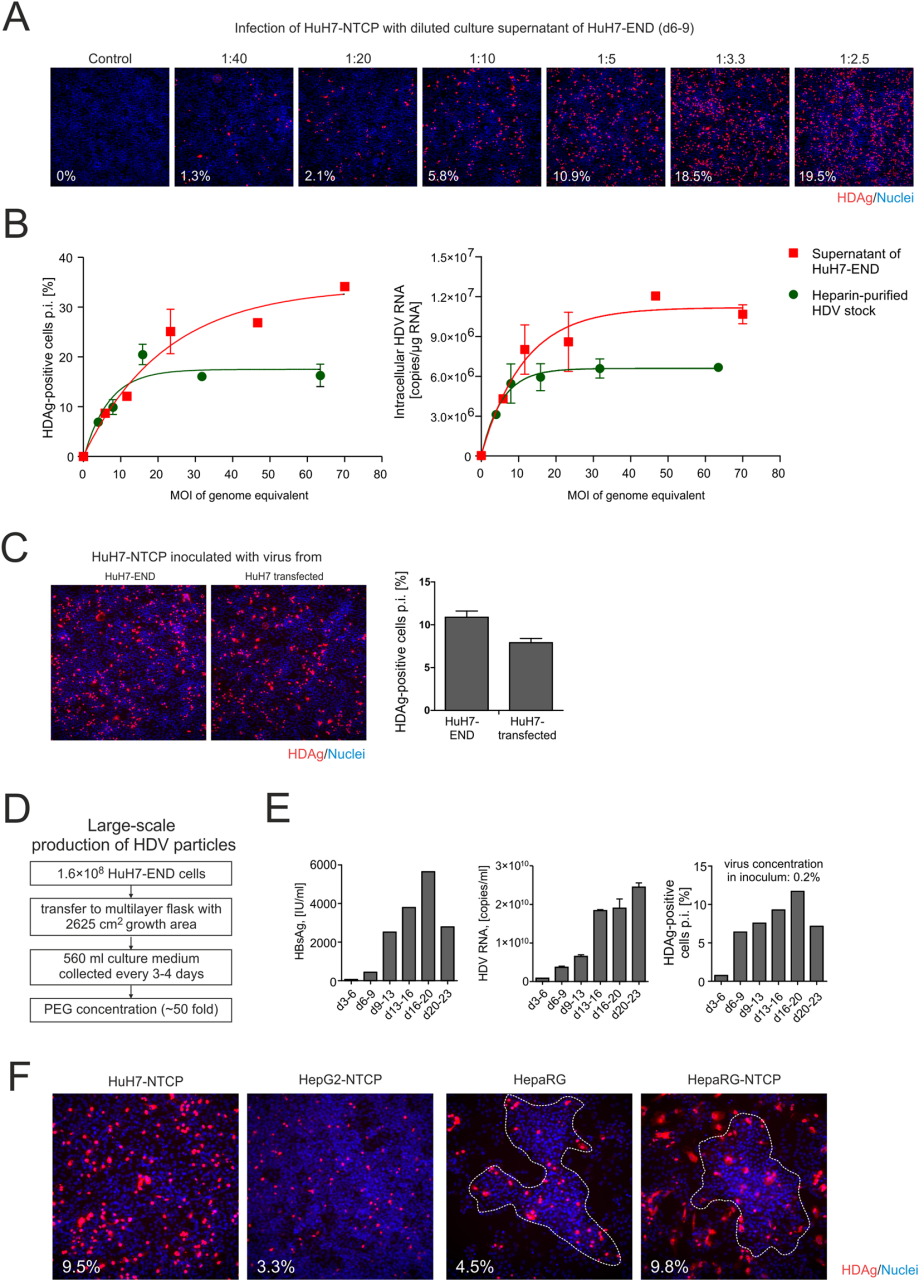
Secretion of infectious HDV particles by HuH7-END cells. (**A**) HDAg-specific IF of HuH7-NTCP cells inoculated with different dilutions (1:40 to 1:2.5) of cell culture supernatants collected from HuH7-END cells between day 6–9 post seeding. HDAg-positive cells after infection were counted and depicted on the left corner of pictures. (**B**) HuH7-NTCP were infected with the culture supernatant of HuH7-END cells or a different source of HDV (heparin-column purified) at different MOI of HDV genome equivalents. The HDAg positive cells were counted (left) and the intracellular HDV RNA (right) were measured at 5 days post-infection. (**C**) Comparison of infectivity of HuH7-END derived HDV with virus obtained from co-transfection of HuH7 cells with pSVLD3 and pT7HB2.7: IF of HDAg in HuH7-NTCP cells after infection with cell culture supernatant of HuH7-END cells (collected between d6-9 post seeding and diluted 1:10) and SN (collected day 10–12 post transfection and diluted at 1:10) Infection rates were quantified by counting HDAg positive HuH7-NTCP cells at day 5 p.i. as shown on the right. (**D**) Scheme of large scale production and concentration of virus stocks produced from HuH7-END cells. (**E**) Characterization of concentrated virus stocks (according to the scheme depicted in **C**) collected at different time periods post seeding. Secreted HBsAg in IU/ml (left), HDV RNA in genome copies/ml (middle) and the infectivity of 1 µl concentrated virus (0.2% of inoculum) in HuH7-NTCP cells in 24-well plate were determined (right). (**F**) Comparative analysis of the infectivity of HuH7-END derived HDV in four HDV susceptible cell lines (HuH7-NTCP, HepG2-NTCP, differentiated HepaRG and differentiated HepaRG-NTCP). The number given in the pictures indicates the percentage of infected cells. The closing dashed lines indicate hepatic islands.

To evaluate the continuous production of infectious HDV by the HuH7-END cell line, we quantified the infectivity of secreted virus over time (Fig. 2A). Cell culture supernatant of HuH7-END cells were collected between d6 and d9 post-seeding, diluted and used for infection of HuH7-NTCP cells. Five days post-infection, HDAg positive cells were counted and quantified. As shown in Fig. 2A, HDAg-positive cells were detected when HuH7-END cell culture supernatants were diluted 40-fold, indicating high levels of virion secretion. The percentage of infected cells increased to approximately 20% (achieved at a 1:3.3 dilution). However, higher concentrations of supernatant did not further increase the number of infected cells. To determine the correlation of MOI and the level of infection (rate of HDAg-positive cells and intracellular HDV RNA), we used serially diluted HuH7-END supernatant for infection and compared the infectivity to a conventionally-prepared HDV stock. This stock was derived from HuH7 cells co-transfected with plasmids pJC126 and pT7HB2.7 and purified by heparin-affinity chromatography. Similar plateauing of the infection rate were observed for both virus preparations (Fig. 2B). Generally, we found good linearity between the HDV RNA titer and infection rates when the MOI was below 15 genome equivalents/cell (which leads to an infection rate of approximately 15%). The observed plateau is consistent with previous results^24^ and may be due to inhibitory factors in the supernatant of HuH7-END cells or a yet-unknown cellular restriction mechanism against HDV infection.

We next directly compared virus production of HuH7-END cells with HuH7 cells that have been co-transfected with plasmids pSVLD3 and pT7HB2.7^20^. As depicted in Fig. 2C supernatants of HuH7-END cells (harvested between d6-9 post seeding) displayed similar infectivity as the supernatants of co-transfected HuH7 cells (harvested between d10-12 post transfection^25^).

To investigate the possibility of large-scale production of HDV, we cultivate HuH7-END cells in a 5-layer Cellstack® culture chambers. 160 million cells in 560 ml culture medium were seeded per chamber. Medium was replaced every 3–4 days until day 23 post-seeding. Viral particles in the culture medium (560 ml) were precipitated overnight by 6% PEG, centrifuged and suspended in 11 ml PBS (Fig. 2D). The concentrated HDV stock was analyzed for HBsAg, HDV RNA and infectivity (Fig. 2E). Consistent with the findings described in Fig. 1D, secreted HDV RNA increased from d3 to d9. However, virus titers continued to increase and reached a plateau of approximately 2E10 copies/ml between d13 to d23. HBsAg also increased until d20 post seeding. Infectivity of the prepared virus increased overtime (Fig. 2E), displaying similar kinetics as the released HDV RNA. When comparing early (d6-9) with later time points (e.g. d16-20), pronounced secretion of infectious virions was observed at early time points when HBsAg levels and secreted HDV RNA were still comparably low. This indicates a higher specific infectivity (e.g. lower amount of non-infectious viral particles) of virus preparations when harvesting at early time points after seeding. Overall, the pooled preparation between d6 to d23 resulted in 55 ml concentrated virus with a mean titer of 1.4E10 virions/ml.

We confirmed that HDV derived from HuH7-END cells is suitable for the infection of HepG2-NTCP cells, differentiated HepaRG cells and HepaRG-NTCP cells. As expected, all these cell lines can be infected with HuH7-END derived HDV (Fig. 2F). HepG2-NTCP cells showed a constrained susceptibility, as described in a previous study^13^. The differentiated HepaRG cells showed an infection pattern preferentially located in hepatocyte-like cells due to the endogenous NTCP expression^26^. As expected, differentiated HepaRG-NTCP cells over-expressing virus receptor were infected at a higher level compared to HepaRG cells. In these cells, infection of biliary cells could also be observed.

### Stability of HuH7-END cells after passaging

**Figure 3:**
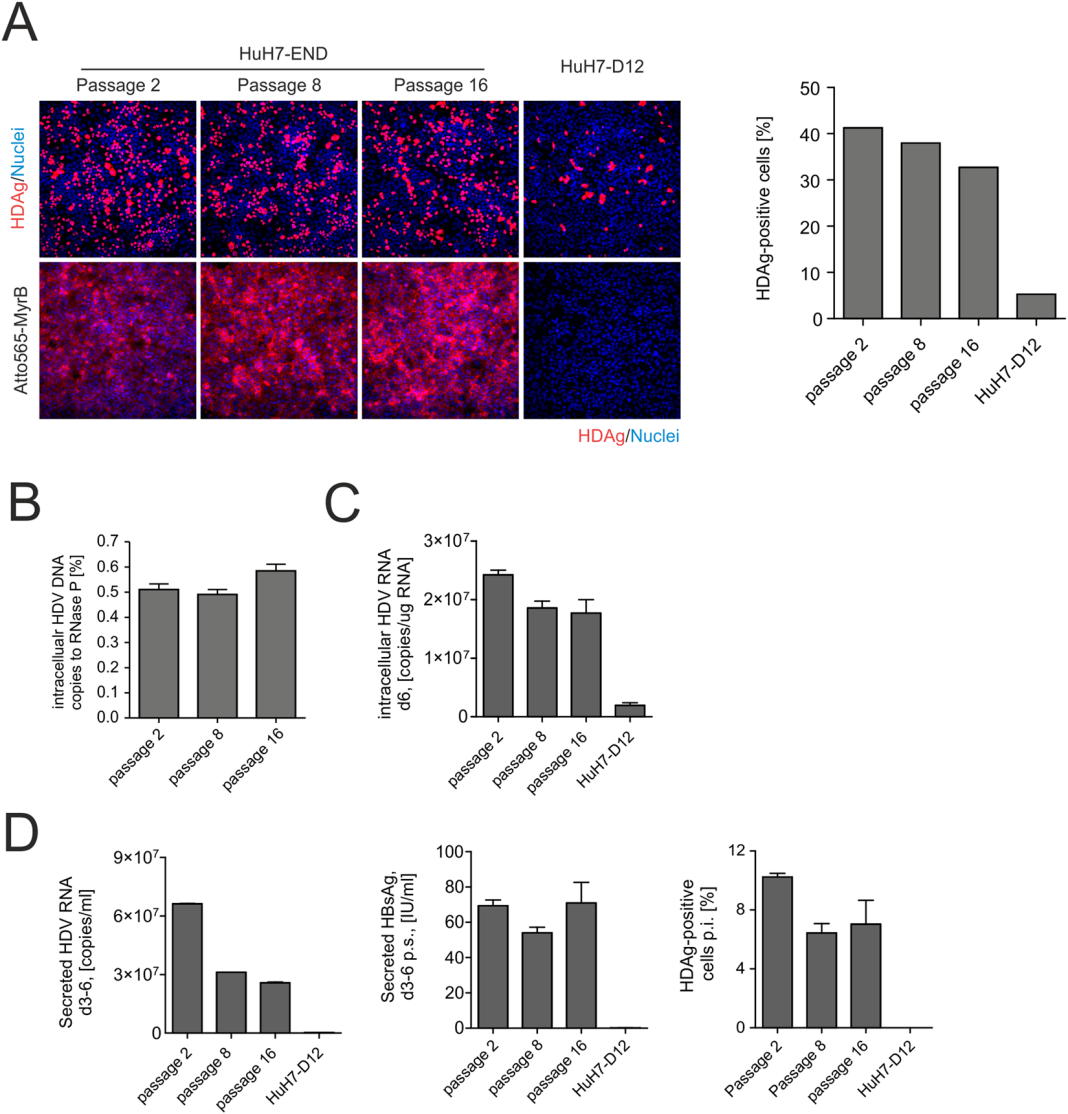
Stability of HDV replication and HDV secretion upon passaging of HuH7-END cells. (**A**) IF staining of HDAg in HuH7-END cells after different passages (passage 2, 8 and 16) in comparison to HDV replicating cell line HuH7-D12 (upper row). In addition, surface NTCP expression (lower row) was quantified using an Atto565-labelled MyrB derivative. The percentage of HDAg-expressing cells is depicted as bars at the right. (**B**) The copy number of intracellular HDV DNA from different passages were quantified by ddPCR in comparison to a single-cope gene RNase P. (**C**) Comparative quantification of intracellular HDV RNA of passage 2, 8 and 16 of HuH7-END versus HuH7-D12 cells. (**D**) Comparative analyses of secreted HDV RNA, HBsAg and infectious HDV in HuH7-END cells at passage 2, 8 and 16 in comparison to HuH7-D12 cells. Note that due to the lack of HBsAg (middle graph) HuH7-D12 cells are negative for secreted HDV RNA (left) and infectious virions (right).

To characterize the selected HuH7-END cell clone with respect to continuity of HDV replication, virus secretion and the stability of the integrated HDV antigenome during cell passaging, we split the cells every 2–3 days and compared intracellular HDV RNA, HDAg expression, copy number of the inserted HDV DNA, secreted HBsAg and RNA, infectivity of released virions of HuH7-END cells at passage 2, 8 and 16. HuH7-D12 cells harboring an integrated cDNA of a trimeric HDV genome was used as a control^27^, As depicted in Fig. 3A, HDAg staining in HuH7-END was comparable between different passages. About 30–40% of cells show strong HDAg expression. In contrast, <5% of HuH7-D12 cells expressed HDAg. DNA copy number analysis of the integrated HDV expression construct showed that there is consistently a single copy of HDV per cell (Fig. 3B). Surface NTCP expression (as evaluated by Atto565-MyrB binding) was also detectable independent of the passage number (Fig. 3A lower panels). Other viral markers (intracellular HDV RNA, secreted HBsAg and secreted infectious particles) remained constant, except for an approximately 2-fold reduction of secreted HDV RNA at higher passage numbers (Fig. 3C,D). These results demonstrate that the HuH7-END cell line stably replicates and secretes HDV for at least 16 passages.

### Co-culture of HuH7-END cells with HDV-susceptible cell lines allows spreading of HDV via the extracellular route

**Figure 4:**
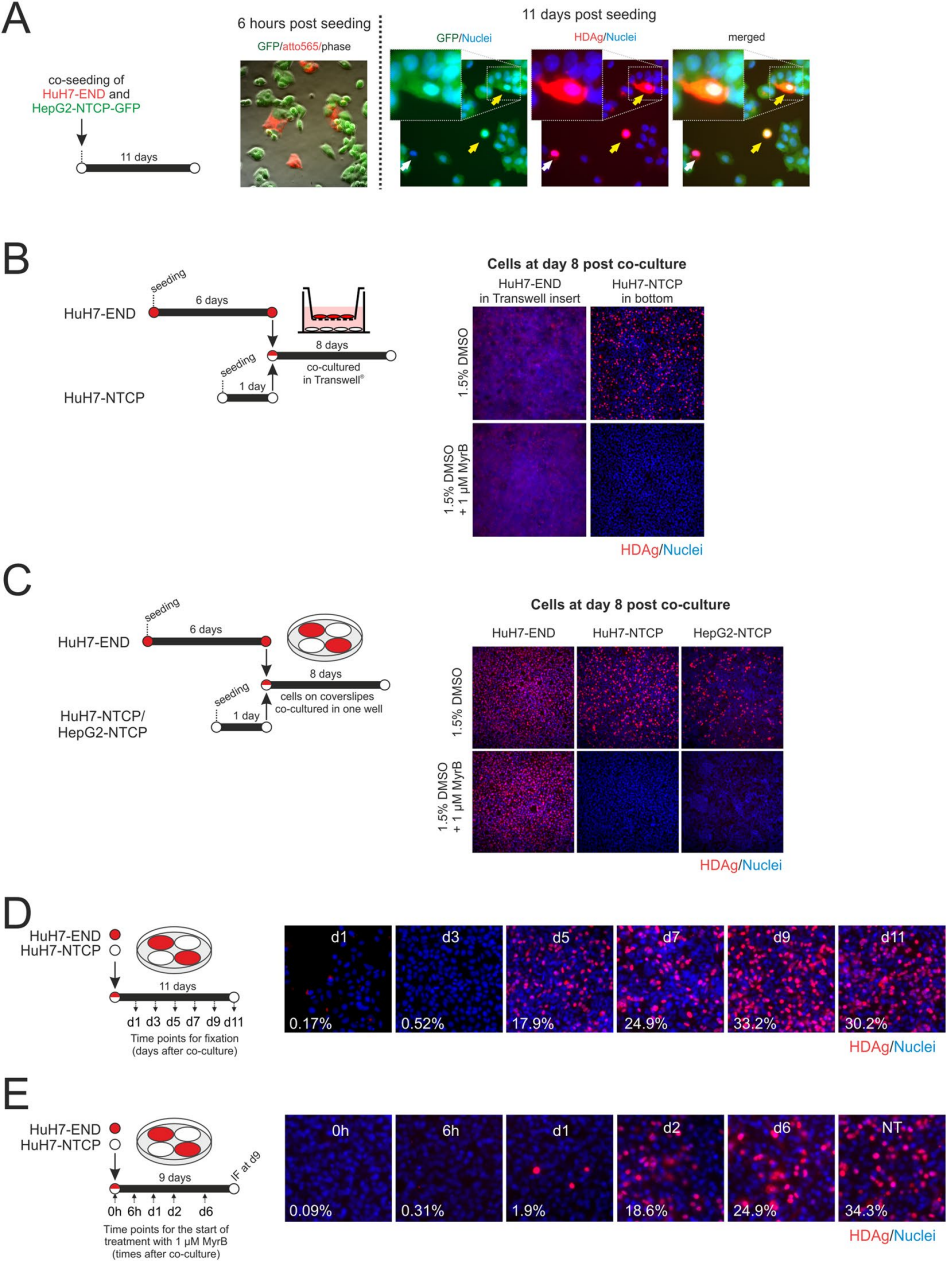
Spread of HDV from HuH7-END to susceptible co-cultured cell lines. (**A**) Co-cultivation of HuH7-END and HepG2-NTCP-GFP cells. HuH7-END cells were temporarily labelled via binding of Atto565-MyrB with NTCP and subsequently co-seeded with HepG2-NTCP-GFP cells (ratio 1:6). 6 hours post seeding, the two cell populations were visualized by GFP fluorescence (green identifying HepG2-NTCP-GFP cells) and Atto565-MyrB fluorescence (red identifying HuH7-END cells). 11 days post co-culture, cells were fixed and stained for HDAg (red) and GFP (green). Cells positive for HDAg (red) identify HuH7-END producer cells (white arrow) while cells positive for both HDAg and GFP (yellow arrows) are infected HepG2-NTCP-GFP cells (zoon-in picture on upper left), indicating that spread occurred. (**B**) Co-cultivation of HuH7-END cells with HuH7-NTCP in Transwell plate. HuH7-END cells grown in a Transwell insert for 6 days were co-cultured with HuH7-NTCP cells seeded on the bottom of well. Medium supplemented with entry inhibitor MyrB were used as a control. Eight days after co-culture, cells were stained for HDAg (red) and nuclei is counterstained by Hoechst (blue). (**C**) Co-cultivation of HuH7-END cells with HuH7-NTCP or HepG2-NTCP cells in coverslips. HuH7-END cells (seeded on coverslips for 6 days) and HuH7-NTCP or HepG2-NTCP cells (seeded on coverslips for 1 day) were co-cultured (left panels) in the presence or absence of the entry inhibitor MyrB. Eight days after co-culture, cells in coverslips were stained for HDAg (red) and nuclei is counterstained by Hoechst (blue) (right panels). (**D**) Kinetics of HDV spread determined by HDAg expression in HuH7-NTCP recipient cells co-cultured with HuH7-END cells. Left, two cell lines are pre-seeded in coverslips as shown in Fig. 4C and co-cultured for 11 days; Right, HDAg staining at different time points after co-seeding as shown by IF of HDAg (red) and nuclei (blue) in the recipient cell line HuH7-NTCP (the number given in the pictures indicates the percentage of infected cells). (**E**) Inhibition of HDV spread by MyrB in time course. Left, MyrB administration scheme (6 h, 1d, 2d, and 6d post co-culture 1 µM MyrB was added and replenished whenever medium was changed; Right, HDAg staining at different time points after co-seeding as shown by IF of HDAg (red) and nuclei (blue) in the recipient cell line HuH7-NTCP (the number given in the pictures indicates the percentage of infected cells).

To investigate whether HDV secreted by HuH7-END cells can spread to surrounding susceptible cells, we co-cultivated HuH7-END cells with HDV-susceptible NTCP-expressing cell lines. In the first experiment, cells were co-cultured with HepG2-NTCP-GFP cells, a stable cell line simultaneously expressing NTCP and GFP (green fluorescent protein). Prior to seeding the HuH7-END donor cells were incubated with 100 nM Atto565-MyrB, which allows specific labelling of the HuH7END cells via surface NTCP. Six hours after co-culture, two populations of cells could be distinguished by fluorescence microscopy (HuH7-END cells in red, labelled by Atto565-MyrB and HepG2-NTCP-GFP cells in green, expressing GFP) (Fig. 4A, left). Eleven days after co-seeding (when membrane-bound Atto565 signal in the HuH7-END cells was degraded), we performed IF staining for HDAg. We detected cells that were positive for both HDAg and GFP. This indicates spread of HDV from HuH7-END to HepG2-NTCP-GFP cells.

In a second experiment, we cultivated HuH7-END cells in Transwell cell culture inserts for 6 days and then transferred the inserts to a well containing HuH7-NTCP cells. After 8 days co-culture, HDAg was detectable in HuH7-NTCP cells, indicating that virus spread across the Transwell occurred. This spread could be efficiently inhibited by MyrB indicating the requirement of *de novo* entry via the HDV receptor NTCP (Fig. 4B).

Finally, we cultivated HuH7-END cells on coverslips for 6 days and subsequently co-cultured them with the two recipient cell lines HuH7-NTCP or HepG2-NTCP. Eight days post co-cultivation, ca. 20% of HuH7-NTCP cells were positive for HDAg (Fig. 4C). In comparison, HepG2-NTCP cells were infected to a lower extent, which is consistent with their lower susceptibility to HDV (Fig. 2E). Supplementing the culture medium with MyrB prevented infection of the recipient cell lines (Fig. 4C, lower panels, right), verifying that spread proceeds by secretion and *de novo* infection of cells. The presence of MyrB did not significantly influence the total number of HDAg-positive HuH7-END cells.

We characterized the kinetics of spread to the recipient cells in a time course experiment. While HDAg-positive HuH7-NTCP cell cells were barely detectable at d3 post seeding, approximately 17% of cells stained positive for HDAg at d5. This number increased and plateaued to approximately 30% at the later points in time (Fig. 4D). Blockade of HDV spread by MyrB at different points in time indicated that spread starts within one day of co-culture (Fig. 4E).

### HuH7-END cells are susceptible to *de novo* HDV infection

**Figure 5:**
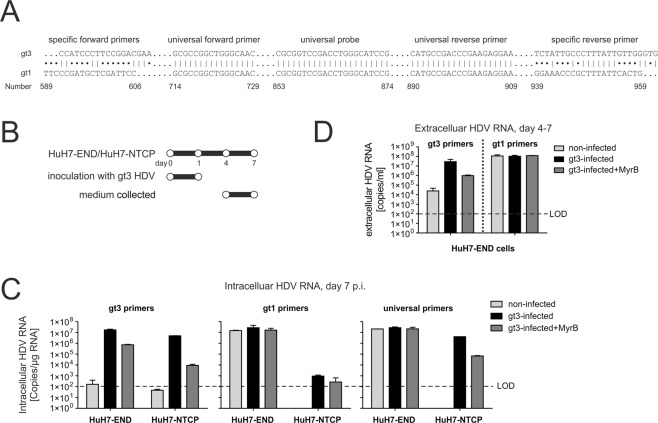
Infection of HuH7-END cells with genotype 3 HDV. (**A**) Alignment of genotype 1 and 3 HDV with respect to the primers used for quantification. The letter shown in the alignment is the binding site of three primer pairs (gt1, gt3 and universal) and the commonly used probe. (**B**) Scheme of HuH7-END cells infected with gt3 HDV (MOI of 300 genome equivalents/cell). HuH7-END cell or HuH7-NTCP cell were inoculated with gt3 HDV overnight. The cell at d7 p.i. were analyzed for intracellular HDV RNA using three primers pairs (**C**) and the culture supernatant between d4-7 post infection were quantified for gt1 or gt3 HDV (**D**).

As shown by Atto-MyrB binding (Fig. 1E), NTCP is expressed on the cell surface of HuH7-END cells. The same cells express and secrete high levels of the receptor ligand (the HBV L-protein), which could principally interfere with NTCP receptor activity. To test the functionality of NTCP for *de novo* receptor mediated entry of HDV, we took advantage of a genotype 3 (gt3) HDV, which can be genetically discriminated by selective PCR from the integrated genotype 1 (gt1) HDV. To this end, two primers pairs binding to non-homologous sequences within the two genotypes were designed. The PCR products produced by genotype-specific primer pairs are larger than the product obtained by the non-discriminating universal primers. All amplicons are detectable with the same probe (Fig. 5A). The selectivity of the genotype specific qPCRs was 10,000-fold for gt1 and 1,000,000-fold for gt3 (Supplemental Fig. 2). HuH7-END and HuH7-NTCP cells were infected with gt3 HDV overnight. After washing, the cell culture supernatants between d4-7 p.i. were collected (Fig. 5B) and intracellular HDV RNA at d7 p.i. were quantified. Approximately 1.7E7 copies of gt3 HDV RNA per µg RNA were detected in HuH7-END cells, which is higher than those of gt3-infected HuH7-NTCP cells (5E6 copies/µg RNA). Importantly, gt3 infection in both cells can be inhibited by MyrB, indicating that NTCP-mediated entry of gt3 HDV had occurred (Fig. 5C left). Compared to gt3, approximately the same amount of gt1 HDV RNA (approximately 2E7 copies/µg RNA) was detected in HuH7-END cells in the presence or absence of gt3 infection (Fig. 5C middle). Finally, we confirmed that gt3 HDV was assembled and secreted into the cell culture medium (Fig. 5D). In summary, we showed that HuH7-END cells allow *de novo* entry and replication of exogenous HDV.

### Drug evaluation using HuH7-END

**Figure 6:**
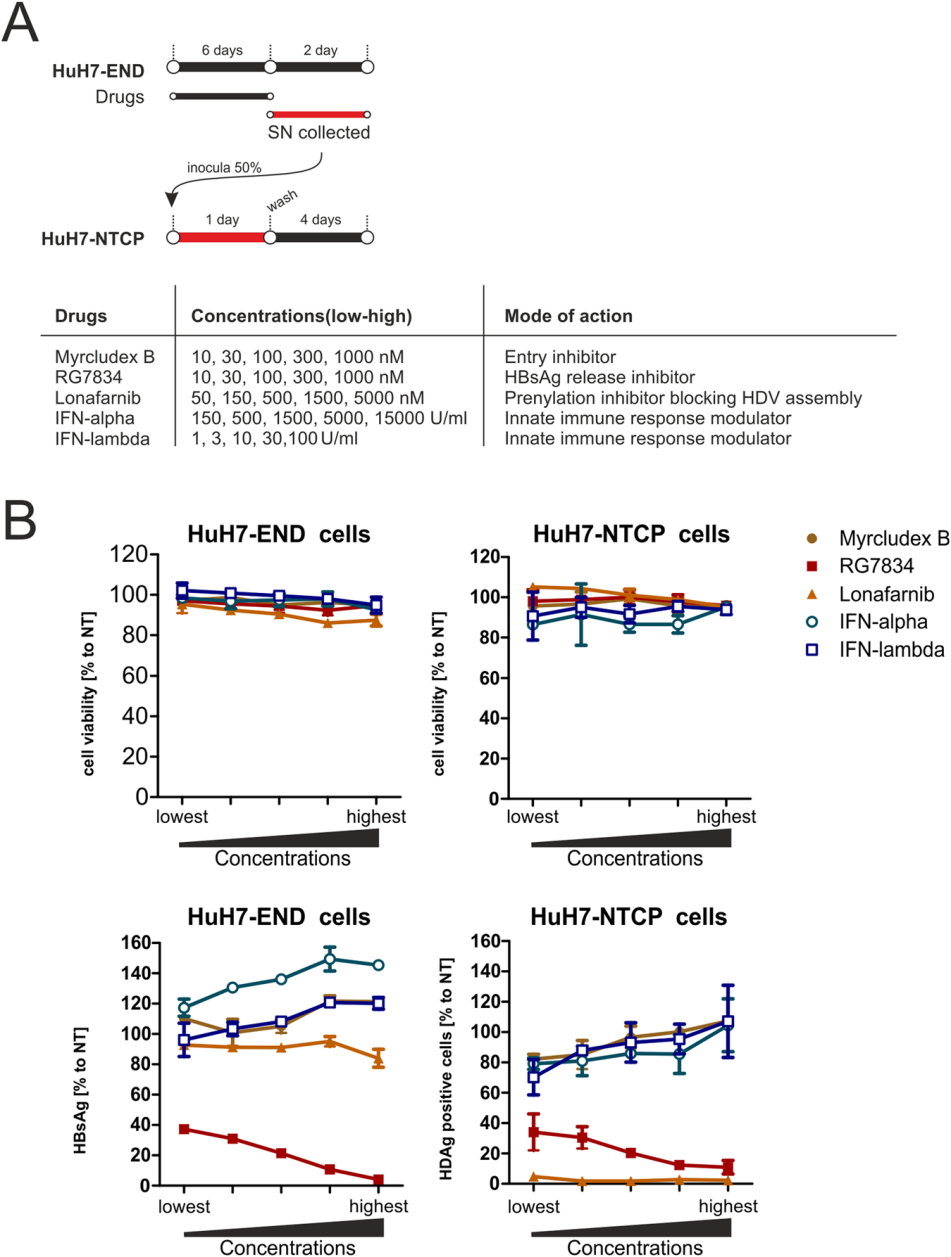
Evaluation of drug efficacy using HuH7-END cells. (**A**) Schematic presentation of a combined drug screening approach using HuH7-END cells in 96-well plate. 5 different substances (MyrB, RG7834, Lonafarnib, IFN-alpha and IFN-lambda) with different modes-of-action (right) were administered for 6 days in HuH7-END cells. Following removal of the drug, cells were cultivated for another 2 days and the culture supernatant was analyzed for HBsAg and used to infect HuH7-NTCP cells. (**B**) Cell viability tested by WST-1 assay (upper panels) and quantification of HBsAg secretion in HuH7-END (lower left) and infectivity assay determined in HuH7-NTCP cells (lower right). NT, non-treated control.

We investigated the effect of five representative drugs with known modes of action on HDV replication using HuH7-END cells. Those are MyrB (an entry inhibitor blocking NTCP), RG7834^28^ (an inhibitor targeting HBV-specific transcripts), Lonafarnib (targeting farnesyl transferase and thereby interfering with HDV particle release), IFN-alpha and IFN-lambda (having pleiotropic effects on HDV replication). (Fig. 6A left). HuH7-END cells were seeded in 96-well plates and treated for 6 days with the different compounds at the indicated concentrations. The culture medium from d6-8 post-treatment (without drugs) were used for a second round infection of HuH7-NTCP cells. Cytotoxic effect at d8 post-treatment were monitored using the WST-1 cell viability assay.

No specific toxicity could be detected even at the highest concentrations applied (Fig. 6B upper panels). The effects on HBsAg secretion (Fig. 6B lower left panel) and on the infectivity of the released HDV (Fig. 6B lower right panel) were determined. As expected from their known mode of action, MyrB and Lonafarnib had no significant effect on HBsAg secretion in HuH7-END cells. Both IFN-alpha and lambda have no detected inhibition. In contrast, the HBV-specific transcription inhibitor RG7834 showed a strong and dose dependent inhibition of HBsAg secretion^28^.

This decrease of HBsAg secretion driven by RG7834 strongly correlated with a decrease in HDV infectivity of the supernatant as seen in the second round of infection. An even more pronounced effect on the release of infectious HDV particles was observed by Lonafarnib treatment, in this case without affecting HBsAg secretion levels, consistent with its known mode of action^29^. MyrB as an entry inhibitor did not affect an already established HDV replication. IFN-alpha and lambda had no significant influence on HDV replication and release as described in previous reports^13,30^.

Taken together, these results demonstrate that drugs targeting multiple steps of the HDV life cycle (including intracellular RNA replication, envelopment and assembly) can be reliably investigated for their mode of action using HuH7-END cells.

## Discussion

This study describes a stable cell line (HuH7-END) that supports continuous and high-level production of infectious HDV particles. The cell line was engineered by step-wise stable introduction of (i) a replication-competent HDV antigenomic cDNA, (ii) a 2.7 kb HBV sub-genomic fragment encoding the three HBV envelope proteins under authentic promotor/enhancer control and (iii) NTCP, the entry receptor of HBV and HDV. Moreover, we demonstrate that HuH7-END cells are suitable for screening antiviral drugs that target late stages of HDV replication directly or indirectly by interfering with HBV envelope protein secretion. We found that HuH7 were highly supportive for virus replication, perhaps because they partially lack innate immune responses restricting replication^13^.

### The stability of HDV replication

HuH7-END cells can be passaged for at least 16 rounds without significant changes in HDAg expression levels, surface NTCP expression or copy number of the integrated HDV expression construct. Moreover, their capability of virus production remained almost unaffected (Fig. 3) indicating a constant HBV envelope protein production. Besides HuH7-END cells, the only stable cell line containing both, integrated HDV genome and the HBV envelope genes, are H1delta9 cells^31^. However, this cell line does not produce HDV for unknown reasons. Freitas *et al*. have shown that PLC/PRF/5 cells after transient transfection with the plasmid pSVLD3 release infectious HDV, although the virus titers in the cell culture supernatant did not exceed 5E5 copies/ml^32^. Since the integrated HBV DNA in H1delta9 cells constantly produce envelope proteins^33^, it is possible that the integrated HDV sequence is replication-competent but defective for assembly. Notably, even when cultured for 6 months (>22 passages), no L-HDAg was detected in H1delta9 cells, suggesting that ADAR-mediated editing of HDV RNA is defective in this system. In contrast, HuH7-END cells continually express L-HDAg. The different kinetics of L-HDAg expression between HuH7-END and H1delta9 cells could be responsible for the differences in HDV particle production.

In HuH7-END cells, continuous expression of L-HDAg does not completely suppress HDV RNA replication. This is consistent with the observation that L-HDAg does not suppress HDV RNA synthesis once replication is established^34^. On the other hand, *de novo* expression of RNA from the integrated template constantly generates HDV templates encoding S-HDAg crucial for replication, thus limiting the effect of continuous error-prone replication via rolling circle mechanisms.

Culture media of HuH7-END cells harvested at late time point (d16-20) had about 6-fold higher RNA titers compared to those harvested at early points in time (d6-9). However the infectivity of these supernatants is only 2-fold higher (Fig. 2). It is possible that virions containing edited genomic RNA (such as L-HDAg encoding RNA) were increased at later time points, which would cause non-productive infections because of the lack of S-HDAg. If the higher specific infectivity rather than total infectivity is desired, we would recommend collecting early culture medium (e.g. d6-9) for virus preparation. The virus titer during this time period ranges from 2E7 (Fig. 1) to 1E8 copies/ml (Fig. 5), a value that is already higher than the average viral load of chronic infected patients^35^.

### Heterogeneity of antigen expression and HDV replication in HuH7-END cells

Even though HuH7-END cells are derived from a single cell clone, only a subpopulation of cells stained strongly positive for HDAg at a given time point. The rest of cells are either HDAg-negative or display a weak punctate staining of HDAg in the nucleus (Fig. 1). A similar pattern has been described before in the HuH7-D12 cell line^21^. In the present study, less than 5% of HuH7-D12 cells stained strongly positive for HDAg (Fig. 3A). It is unclear, why only a subpopulation of a stable cell clone express HDAg at a given time point. It is also unknown if these “inactive” HDAg-negative cells are permanently silenced for HDV replication and HDAg expression or whether they undergo a dynamic change in activation and silencing over time.

Almost all cells of the parental HuH7-HDV cell line were positive for HDAg early after G418 selection. However, after several rounds of cell passaging, the number of HDAg-negative cells increased. This could be due to either the genomic instability of HuH7 cells or shutdown of HDV RNA replication for an unknown reason. To test for a possible genetic instability, we serially diluted the selected HuH7-END cell clone and performed an additional round of clonal isolation. 10 single cell colonies were isolated and analyzed for HDAg (Supplemental Fig. 3). HDAg was detected in all of them except one clone. Among them, 6 clones (C2, C4, C5, C6, C7 and C8) displayed strong HDAg in nuclei, and 3 clones (C1, C3 and C9) mainly displayed speckle like HDAg. It indicates that the majority of HuH7-END cells (at least 90%) can replicate HDV and therefore argues against the loss of the encoding integrate. This result is also consistent with the stability of the integrated construct following multiple cell passages (Fig. 3B).

### Role of NTCP

Expression of NTCP on the surface of HuH7-END cells permitted superinfection with genotype 3 HDV virions (Fig. 5). This implies the possibility that cells may generally allow re-entry of secreted HDV to boost intracellular HDV replication by an “autocrine” loop. However, as shown in Fig. 4B, the presence of MyrB in the cell culture for 8 days had no significant effects on the number of HDAg positive HuH7-END cells, indicating that HDAg expression in these cells does not require re-entry. Moreover, a 6-day treatment with MyrB did not reduce the secretion of infectious virus significantly (Fig. 6). We therefore concluded that the surface NTCP does not help HDV replication during that time. Notably, in these experiments, the duration of MyrB treatment are still short in comparison to the virus peak coming at d18-21 post seeding. It is still possible that NTCP change the kinetics of virion production at these later time points.

## Materials and Methods

### Chemicals

Lonafarnib was purchased from MedChem Express. IFN-alpha (2a) and lambda was purchased from PBL and PeproTech respectively. RG7834 analogue was synthesized according to the chemical structure described^28^.

### Plasmids

Plasmid pJC126 (genotype 1) containing a 1.1-fold cDNA copy of the HDV antigenome was kindly provided by John Taylor^19^. Plasmid pcDNA3.1-HDV-gt3-peru containing genotype 3 HDV antigenome similar to pJC126 was generated by inserting a synthetic 1.1- HDV antisense sequence (Accession number L22063, gene synthesized by Eurofins Genomics, Ebersberg, Germany) into the *Hind*III/*Eco*RI restriction sites of the plasmid pcDNA3.1 Zeo(+). Plasmid pSVLD3 harboring a trimer of the HDV gt1 genome (accession number M21012.1) was also provided by John Taylor and pT7HB2.7 encoding the HBV envelope proteins was a gift from Camille Sureau^20^. The plasmid pWPI-NTCP^11^, allows production of a lentiviral vector encoding NTCP and harbors a puromycin resistance gene. pWPI-NTCP-GFP was constructed by replacing the puromycin resistance gene GFP. Plasmid pLX304-HB2.7 is a lentiviral vector expressing HBV envelope proteins^16^, which was constructed by insertion of the HBV sequence from pT7HB2.7 into the lentiviral vector pLX304^36^. The sequence of all constructs was verified by Sanger sequencing (GATC Biotech).

### Lentivirus

For production of lentiviruses encoding NTCP or the HBV envelope proteins^11^, HEK-293 cells were co-transfected with pWPI-NTCP or pLX304-HB2.7 and the two plasmids pMD2.G and psPAX2 (a gift from Didier Trono) using Mirus TransIT LT1 transfection reagent (Mirus, Germany). The supernatants containing the respective lentiviral pseudoparticles were harvested between 12 h and 36 h post transfection, filtered through a 0.45 μm filter, and concentrated by ultracentrifugation at 20,000 rpm (SW28 rotor) for 2 hours at 4 °C. The precipitated lentiviral particles were suspended in DMEM and used immediately or stored at −80 °C. For establishment of stable cell lines, cells one day post seeding with 70% confluence were inoculated with lentivirus in the presence of 4% polyethylene glycol (PEG, Mw 8000). Three days after transduction, 5 µg/ml puromycin (for pWPI-NTCP) or 20 µg/ml blasticidin (for pLX304-HB2.7) were added to medium to select for stably-transduced cells. Generally, 90% of cells survived the selection without obvious morphological change compared to the untransduced cells.

### Cells

For generation of HuH7-END cell line, HuH7 cells were transfected with pJC126 using Mirus TransIT LT1 transfection reagent (Mirus, Germany) and then selected with 1 mg/ml G418. The resulting HuH7-HDV cells were transduced with lentivirus pLX304-HB2.7, selected with 20 µg/ml blasticidin, transduced with pWPI-NTCP, selected again with 5 µg/ml puromycin. The resulting HuH7-HDV-Env-NTCP cells were serially diluted to grow single cell colonies. The cell clone B1 was named as HuH7-END.

HuH7-D12 cells were obtained from Sigma (Cat. 01042712-1VL). HuH7-NTCP and HepG2-NTCP are cell lines expressing human NTCP as described previously^11^. HepG2-NTCP-GFP is a selected cell clone expressing both NTCP and GFP after transduction with lentiviral vector pWPI-NTCP-GFP^11^. All these cell lines were cultivated in DMEM supplemented with 10% fetal calf serum, 2 mM l-glutamine, penicillin (50 U/mL), and streptomycin (50 μg/mL). HuH7-END cells were cultivated in the same medium with additional 2.5 μg/ml puromycin, 500 µg/ml G418, and 10 µg/ml blasticidin. Cultivation and differentiation of HepaRG or HepaRG-NTCP cells were performed as described previously^11^.

### HDV production in HuH7-END cells

For production of HDV or determination of viral kinetics, HuH7-END cells were seeded at density of 2.5E5 cells/cm^2^ in DMEM medium supplemented with 10% fetal calf serum, 2 mM l-glutamine, penicillin (50 U/mL), streptomycin (50 μg/mL) and 0.5% DMSO (dimethyl sulfoxide). The same medium was used for further cultivation and medium was changed every 3 days if not indicated otherwise.

### Preparation of gt3 HDV

Plasmid pcDNA3.1-HDV-gt3-peru and pT7HB2.7 were used to transfect HuH7 cells using Mirus TransIT LT1 transfection reagent (Mirus, Germany) according to the manufactory’s manual. The supernatant between day 6–12 post transfection was purified and concentrated using Heparin HP column (GE Healthcare) as described^37^.

### Quantification of HDV genome

Intracellular RNA were exacted by NucleoSpin RNA (Macherey-Nagel) from cell pellets. Extracellular RNA from 140 µL cell culture medium were extracted by QIAGEN QIAmp Viral RNA Mini Kit (Qiagen, Germany). For measuring Glyceraldehyde-3-phosphate dehydrogenase (GAPDH) RNA, high-capacity cDNA reverse transcription kit (ThermoFisher) and iTaq™ Universal SYBR^®^ Green Supermix were used. Input RNA were normalized by GAPDH, which was detected using the following primers: GAPDH-for: 5′-ACCCAGAAGACTGTGGATGG, GAPDH-rev: 5′-TCTAGACGGCAGGTCAGGTC. PCR (polymerase chain reaction) were performed on Bio-Rad CFX96 Touch™ system using the following program: 95 °C (3 minutes), 95 °C (10 seconds), 60 °C (30 seconds) with 40 repeating cycles for the last two steps.

For HDV RNA quantification, intracellular or extracellular RNA were reverse transcribed and amplified using Quanta qScript™ XLT One-Step RT-qPCR ToughMix (Quantabio, Germany) according to the manufacturer’s instructions. The following primers and probe were used for HDV quantification: HDV-for: 5′-GCGCCGGCYGGGCAAC; HDV-rev: TTCCTCTTCGGGTCGGCATG; HDV-Probe: 5′FAM-CGCGGTCCGACCTGGGCATCCG-3′TAMRA^38^. Purified plasmid pJC126 and pcDNA3.1-HDV-gt3-peru were used to prepare the standard for gt1 and gt3 HDV respectively. Reactions were performed on Bio-Rad CFX96 Touch™ system using the following program: 50 °C (20 minutes), 95 °C (60 seconds), 95 °C (10 seconds), and 72 °C (60 seconds) with 40 repeating cycles for the last two steps.

For HDV DNA copy number analysis, total cellular DNA was extracted from cells harvested from 12-well plates using a NucleoSpin® Tissue kit (740952, Macherey-Nagel, Düren, Germany) as per the manufacturer’s instructions and eluted in 50 µL of elution buffer. 5 µL of DNA extract was digested using *Eco*RI-HF (NEB, R3101S) as per the manufacturer’s instructions. 2 µL of digested DNA was placed directly into a 20 µL ddPCR (digital droplet PCR) reaction containing composed of 1x ddPCR Supermix for Probes (1863010, Biorad, Hercules, CA USA), 1x VIC-labelled TaqMan™ Copy Number Reference Assay for the human RNase P gene (4403328, Applied Biosystems, Foster City, CA USA), and 150 pmol of each HDV-specific primer and probe (same as HDV RNA quantification protocol). Droplets were generated according to the manufacturer’s protocol using a QX200 Droplet Generator (Biorad). Intra-droplet PCR was carried out using the following protocol: an initial 10 min denaturation, enzyme activation and droplet stabilization step at 95 °C; followed by 40 cycles of a 10 s denaturation step at 95 °C, a 15 s annealing step at 54 °C and a 20 s elongation step at 68 °C, finished with a 10 minute enzyme deactivation step at 95 °C. Products were then stored at 12 °C until droplet reading using a QX200 Droplet Reader (Biorad), quantification using FAM and VIC channels, and data analysis using QuantaSoft (Biorad).

For genotype specific PCR, primer gt1-for: 5′-TTCCCGATGCTCGATTCC and gt1-rev: 5′-CAGTGAATAAAGCGGGTTTCC were used to detect gt1 HDV; Peru-for: 5′-CCATCCCTTCCGGACGAA and Peru-rev: 5′-CACCCAACAATAAAGGGCAATAGA were used to detect gt3. The probe and PCR program were the same as mentioned above.

### Peptides

Myrcludex B (MyrB) is a myristoylated peptide mimicking the N-terminus of HBV L protein^39^. Synthesis of MyrB was performed by solid phase synthesis. Labelling was achieved by coupling Atto565-NHS-ester (ATTO-TEC, Germany) to the lysine residues of the peptides.

### Immunofluorescence microscopy

For surface NTCP staining, cells grown on coverslips were incubated with 100 nM Atto565-MyrB and 1 µg/ml Hoechst for 20 min. Then cells were washed 3 times with PBS (phosphate buffered saline) and fixed with 4% paraformaldehyde (room temperature, 10 min). Cells were washed again 3 times with PBS before microscopy. For blocking control, cells were pre-incubated with 2 µM MyrB for 20 min and the same concentration of MyrB was added during the incubation with Atto565-MyrB.

For IF staining, cells were fixed with 4% paraformaldehyde for 10 min at room temperature, permeabilized with 0.5% v/v Triton X-100 (room temperature, 10 min) and then incubated with antibodies diluted in 2% BSA. The primary antibody against HDAg is a characterized patient serum (GEAO or VUDA). The monoclonal antibody MA18/7 against DPAF linear motif was used to detect HBV L protein (kindly provided by Wolfram H. Gerlich). As secondary antibodies goat anti-rabbit or anti-human, labelled with either AlexaFluor488 or AlexaFluor546 (Invitrogen) were used. Images were taken on Leica DM IRB or Leica SP8 confocal microscope (Leica, Germany). Image analysis was performed using ImageJ. The plugin “Nucleus Counter” was used for quantification of nuclei and HDAg, since HDAg is mostly located within cell nuclei.

### Quantification of HDV infectivity

To determine the infectivity of HDV, HuH7-NTCP cells were seeded in 24-well plates (2.5E5 cells/well). One day after seeding, cells were infected with HDV in the presence of 4% PEG8000 (Sigma-Aldrich) and 2.5% v/v DMSO. Cells were washed 3 times with PBS on day 1 after infection, and keep cultivated in medium containing 2.5% v/v DMSO. IF against HDAg was performed at d5 post infection. The number of HDAg-positive cells and the total nuclei were counted using software ImageJ with a plugin “Nucleus Counter”. The percentage of HDAg-positive cells to total cell numbers was calculated as infection rate in this study. Notably, due to a saturation, the infection rate under 15% is suitable for quantitative comparisons.

### Co-culture for virus spread

For direct co-culture of HuH7-END with HepG2-NTCP-GFP, HuH7-END were first stained for surface NTCP with 100 nM Atto565-MyrB. Cell mixtures were seeded at 2.5E5 cells/well in 24-well plates, and keep cultivated at culture medium for 11 days.

For co-culture of HuH7-END with HepG2-NTCP or HuH7-NTCP cells, we first grew HuH7-END cells in cover slips for 6 days, HepG2-NTCP or HuH7-NTCP for 1 day. Then two coverslips of HuH7-END cells and HepG2/HuH7-NTCP were put together in one well of 6-well plate. During co-culture, medium containing 1.5% v/v DMSO was used.

### Screening

HuH7-END cells in 96-well plate were cultured in medium with 0.5% v/v DMSO and compounds for 6 days. Then cells were washed 3 times with PBS, and fresh medium with 0.5% v/v DMSO but without compound were added for another 2 days. HuH7-END at day 8 post seeding were determined for cell viability by WST-1 assay. Afterward, the two-day drug-free culture medium were used to inoculate HuH7-NTCP cells. HuH7-NTCP cells at day 5 post infection were determined for cell viability by WST-1 assay. The secreted HBsAg by HuH7-END were quantified by ELISA and the infectious virus were determined by IF against HDAg of inoculated HuH7-NTCP cells.

### HBsAg quantification

HBsAg was quantified by Architect assay (Abbott, Germany). For screening, home-made ELISA were used as described previously^40^.

## Supplementary information

supplementary 1
