# Supplementary material for: Generation and characterization of a stable cell line persistently replicating and secreting the human hepatitis delta virus
Source: Sci Rep. 2019 Jul 10;9:10021. doi: 10.1038/s41598-019-46493-1 (PMC6620269; doi:10.1038/s41598-019-46493-1)

**Supplementary information**

**Generation and characterization of a stable cell line persistently replicating and secreting the human hepatitis delta virus**

Yi Ni^1, 2^, Zhenfeng Zhang^1^, Lisa Engelskircher^1^, Georg Verch^1^, Thomas Tu^1^, Florian A. Lempp^1,2^, Stephan Urban^1,2^

^1^ Dept. of Infectious Diseases, Molecular Virology, University Hospital Heidelberg, Heidelberg, Germany.

^2^ German Center for Infection Research (DZIF), partner site Heidelberg, TTU Hepatitis, Heidelberg, Germany.

**Supplemental figures and legends**

**Supplemental figure 1. Characterization of HuH7-HDV-Env cells** (A) HBsAg secretion of three cell lines. HuH7, HuH7-HDV and HuH7-HDV-Env cells were seeded and the cell culture medium between d2-5 post seeding were analyzed for HBsAg. (B) Secretion of infectious virus by HuH7-HDV-Env. The cell culture supernatant of HuH7-HDV-Env at d0-3 post seeding were used to infect HepG2-NTCP cells. At d5 post infection, HepG2-NTCP were stained for HDAg (red). Nuclei were counter-stained with Hoechst (blue).

**Supplemental figure 2. Primer specificity for the detection of gt1 and gt3 HDV.** For quantitative PCR, 10^8^ copies of plasmids pJC126 (gt1) and pcDNA3.1-HDV-gt3-peru (gt3) were amplified with gt1 or gt3-specific primers. The absolute amount of HDV RNA were calculated using the respective plasmids as standards. The detection limit is around 100 copies/PCR reaction.

**Supplemental figure 3. HDAg expression of 10 clones derived from HuH7-HDV-Env cells** 10 clones (C1-C10) were generated by serial dilution using HuH7-END as the parental cells. These clones were analyzed for HDAg expression (red) by IF.

Supplemental figure 1.


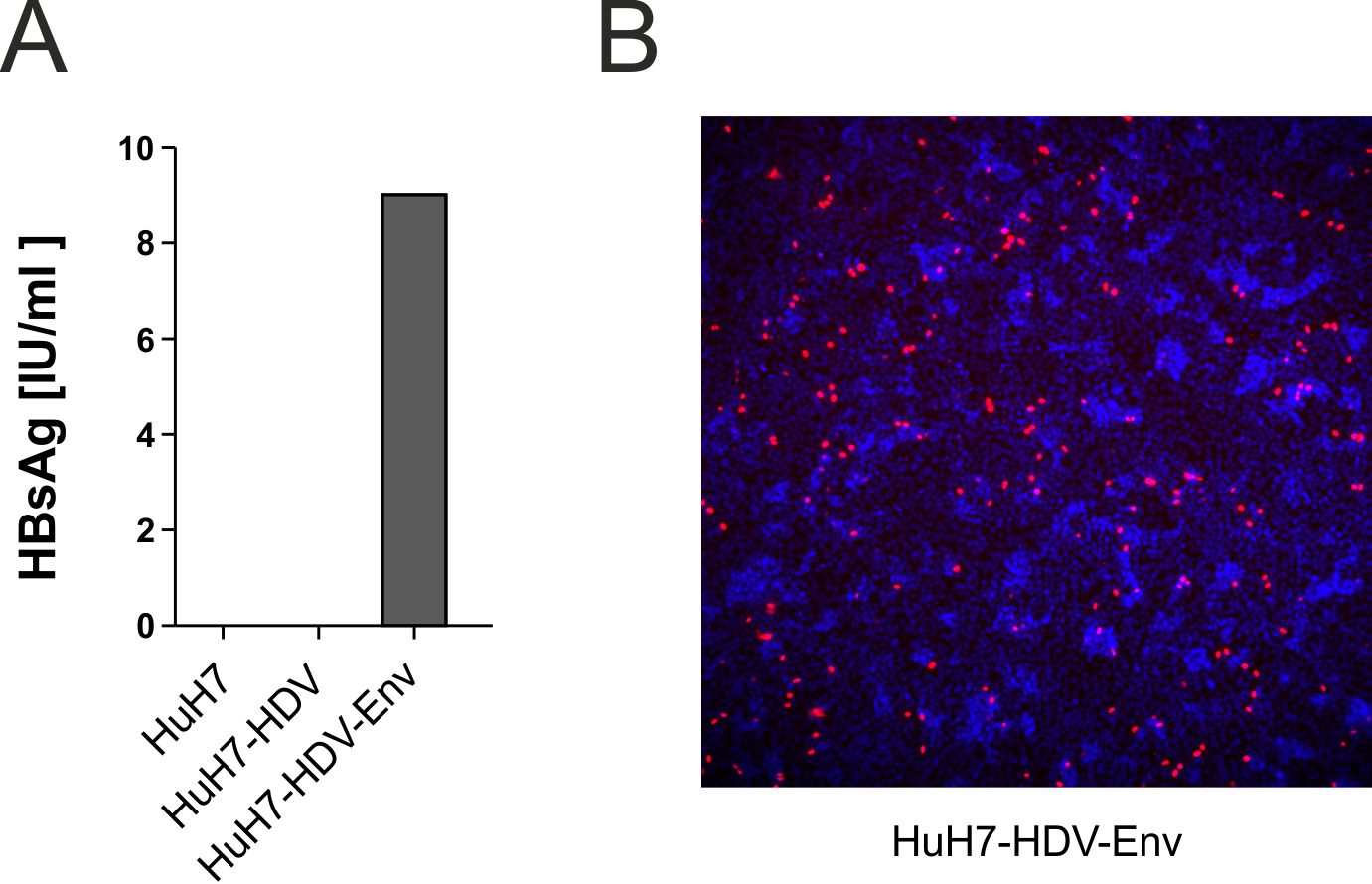


Supplemental figure 2.


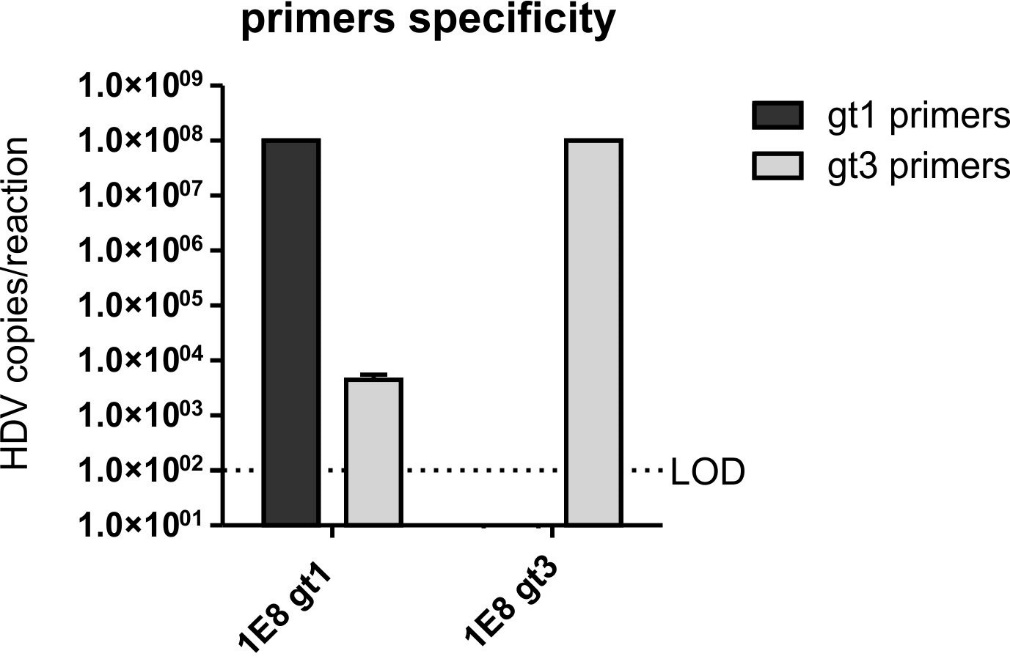


Supplemental figure 3.


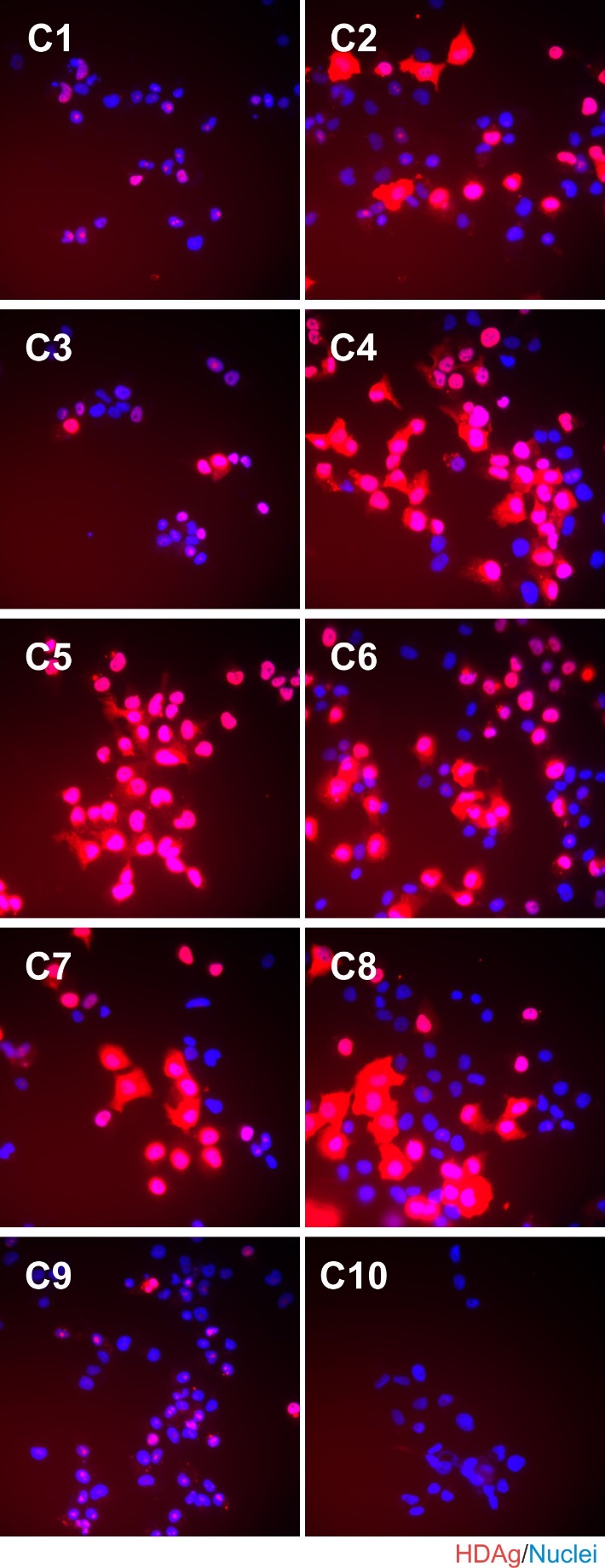


Figure 1C. Files with full-length blot


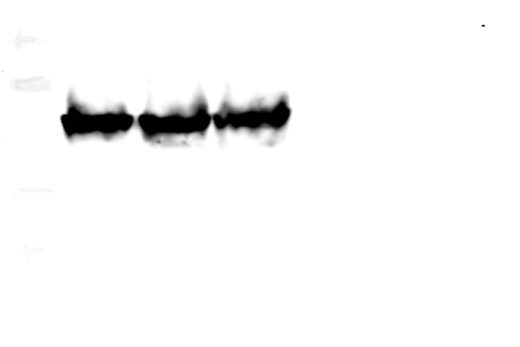


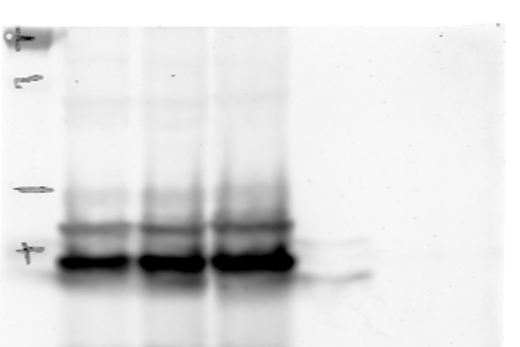

Supplement: Supplementary file 1 — supplementary 1 [file 41598_2019_46493_MOESM1_ESM.docx]
